# Supplementary material for: Increased Indian Ocean-North Atlantic Ocean warming chain under greenhouse warming
Source: Nat Commun. 2022 Jul 8;13:3978. doi: 10.1038/s41467-022-31676-8 (PMC9270466; doi:10.1038/s41467-022-31676-8)
Supplement: Supplementary file 1 — Supplementary Information [file 41467_2022_31676_MOESM1_ESM.docx]

Supplementary material for “Increased Indian-North Atlantic Ocean Warming Chain under greenhouse warming”

Young-Min Yang^1,2^, Jae-Heung Park^3^, Soon-Il An^4*^, Sang-Wook Yeh^5^, Zhiwei Zhu^1^, Fei Liu^6^, Juan Li^1^, June-Yi Lee^7,8^ and Bin Wang^1,9^

^1^ Key Laboratory of Meteorological Disaster, Ministry of Education (KLME)/Joint International Research Laboratory of Climate and Environment Change (ILCEC)/Collaborative Innovation Center on Forecast and Evaluation of Meteorological Disasters (CIC-FEMD), Nanjing University of Information Science and Technology, Nanjing, 210044, China. ^2^State Key Laboratory of Numerical Modeling for Atmospheric Sciences and Geophysical Fluid Dynamics, Institute of Atmospheric Physics, Chinese Academy of Sciences, Beijing 100029, China ^3^Division of Environmental Science and Engineering, Pohang University of Science and Technology, Pohang 37673, Korea. ^4^Department of Atmospheric Sciences and Irreversible Climate Change Research Center, Yonsei University, Seoul 03722, Korea. ^5^Department Marine Sciences and Convergent Technology, Hanyang University, ERICA, South Korea. ^6^School of Atmospheric Sciences Sun Yat-Sen University, Key Laboratory of Tropical Atmosphere-Ocean System Ministry of Education, and Southern Marine Science and Engineering Guangdong Laboratory, Zhuhai 519082, China. ^7^Research Center for Climate Sciences, Pusan National University, Busan, South Korea, ^8^Center for Climate Physics, Institute for Basic Science, Busan, South Korea. ^9^Department of Atmospheric Sciences and International Pacific Research Center, University of Hawaii, Honolulu HI 96822, USA. email: sian@yonsei.ac.kr


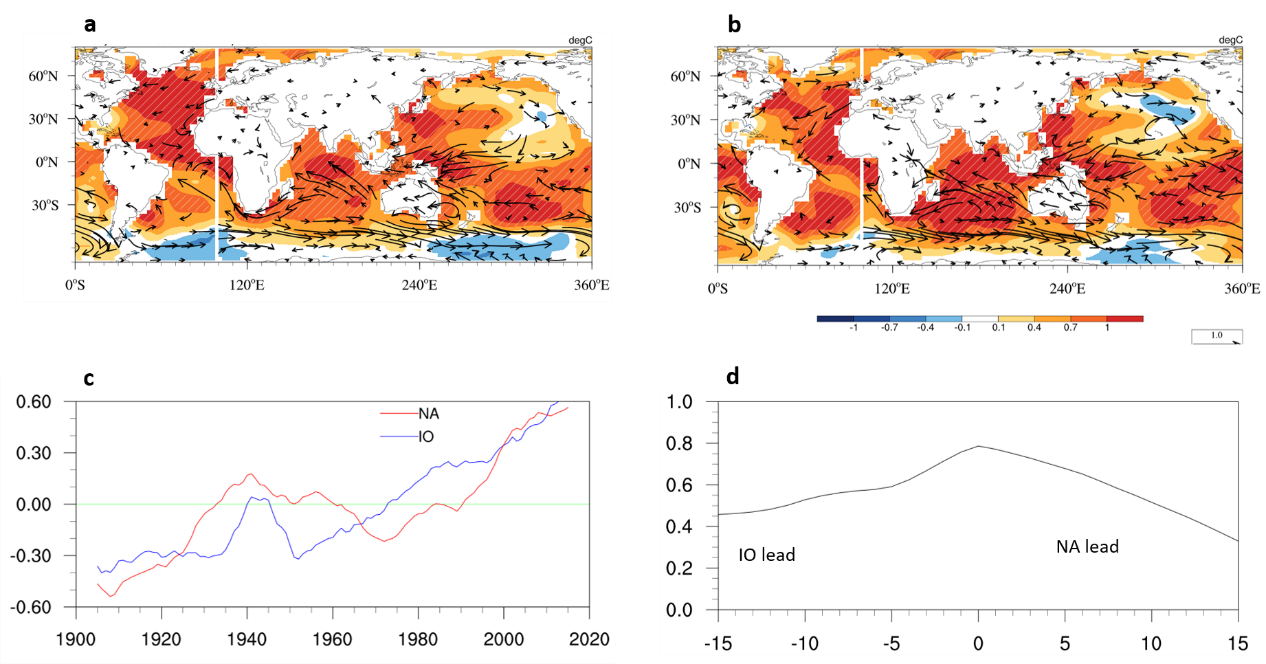


**Supplementary Figure 1 | Historical warming trends. a** Observed SST (K) and surface wind anomalies (arrow, m/s) regressed onto the NA index (0°–70°N, 80°–0°W). The hatched area represents the regressed SSTA and is significant at the 95% confidence level. **b** Same as (**a)** but for the IO index (30S°–30°N, 40°–120°E), **c** Observed time series of the NA (red line) and IO (blue line) indices. The 11-year running average data were used for 1950–2020. **d** Observed lead-lag correlation coefficient between the IO and NA indices. The lag is positive (negative) when the IO leads (lags). The last 5 years were excluded from the analysis. The 11-year running average data were used for 1950–2020.


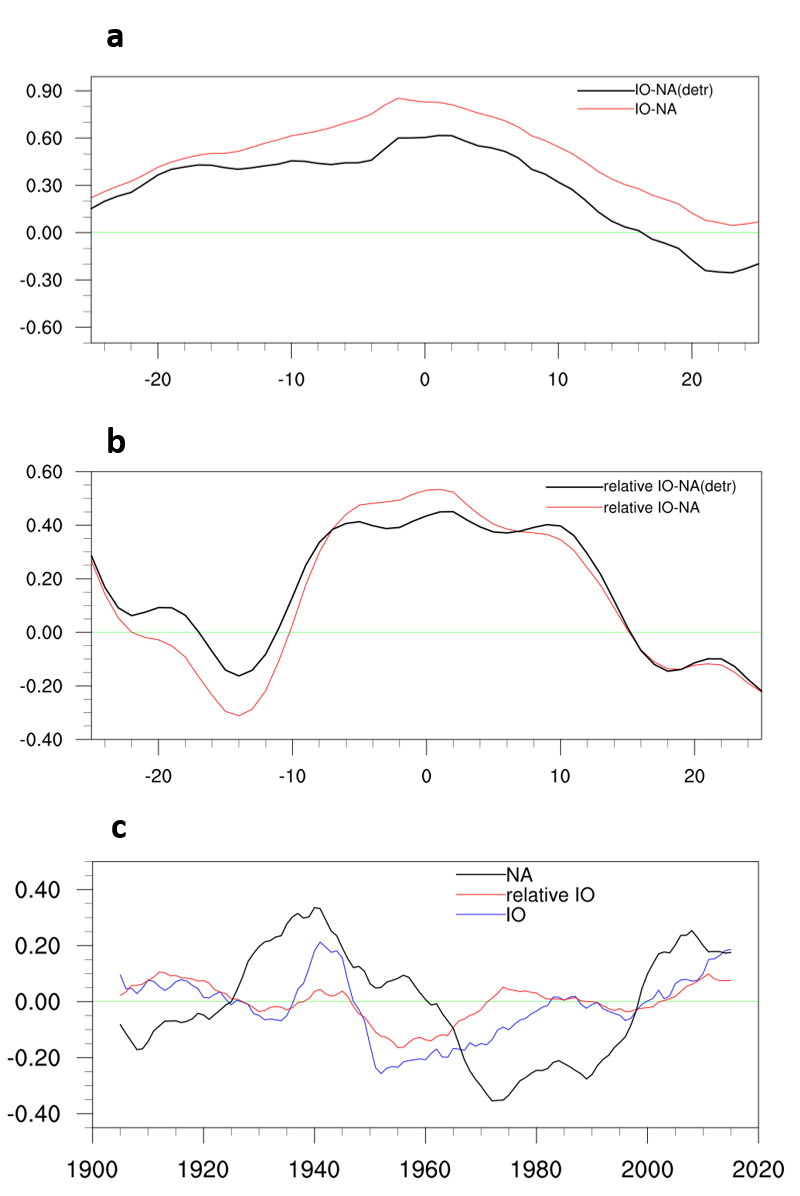


**Supplementary Figure 2 | Relationship between Indian Ocean (IO) and North Atlantic Ocean (IO). a** Observed lead-lag correlation coefficient between the IO and NA indices during 1900-2020. The lag is positive (negative) when the IO leads (lags). The black line represents SST data that are removed long-term linear trends and the red line shows original SST data. **b** Observed lead-lag correlation coefficient between the relative IO and NA indices during 1950-2020. The relative IO and NA are defined as the average SST in the Indian Ocean (30°S–30°N, 40°E–120°E) minus the whole tropical ocean (30°S–30°N, 0-360E). The black line represents SST data that are removed long-term linear trends and the red line shows original SST data. **c** Observed time series of the NA (black line), IO (blue line), and relative IO (red line) indices. For **a** and **b**, long-term linear trends in the SST data were removed before analysis in the black line and the last 5 years were excluded from the analysis.


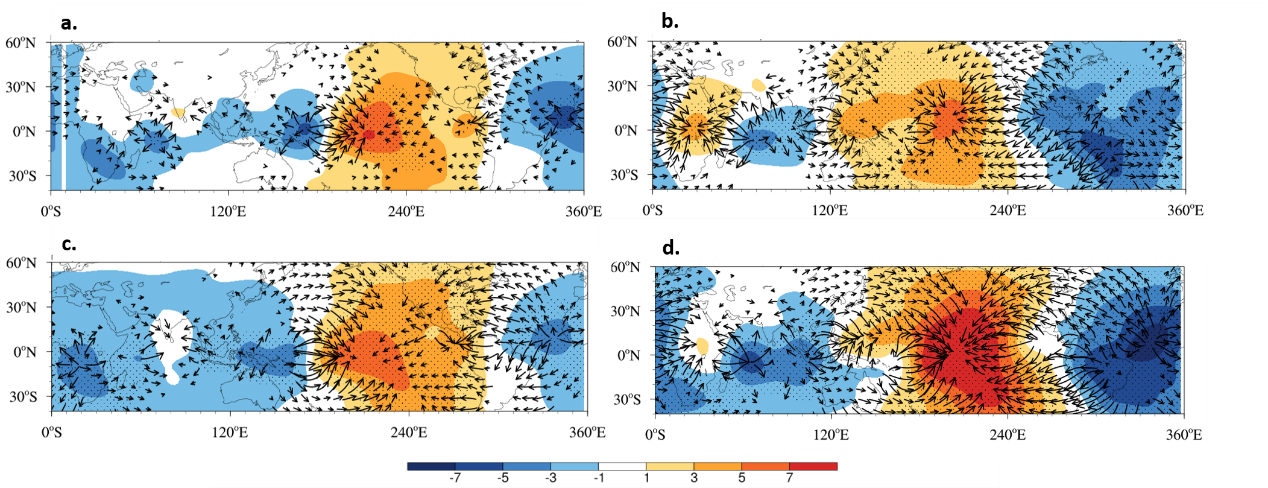


**Supplementary Figure 3 | Upper-level circulation induced by Indian Ocean (IO) and North Atlantic Ocean (NA) warming. a** Observed upper-level velocity potential (shading, 10^5^ m^2^ s^-1^) and divergent winds (m s^-1^) anomalies regressed onto the NA index (0°–70°N, 80°W–0°). The 11-year running average was used for 1950–2019, and the first and last 5 years were excluded. The dotted area represents significant velocity potential at the 95% confidence level. **b** Same as (**a)** but associated with the IO index (35S°–30°N, 40°–120°E). **c** Same as but from the model with observed NA index. **d** Same as but (**b)** from the model with IO index. The model in this study was integrated from 1900 to 2020 with historical external forcings and 71-year data (1950-2020) are utilized for the analysis. Long-term linear trends in the SST data were removed before regression.

**
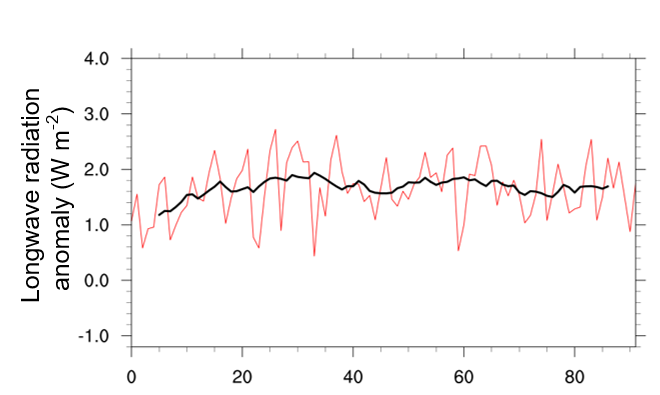
**

**Supplementary Figure 4 |** **North Atlantic Ocean (NA) response to Indian Ocean (IO) warming**. **a** Temporal evolution in annual-mean longwave radiation anomalies between “PI_IO+0C” and “PI_IO+1C” model experiments. The downward direction is positive (warming). The red line shows annual mean data and the black line represents an 11-year running mean of the red line.


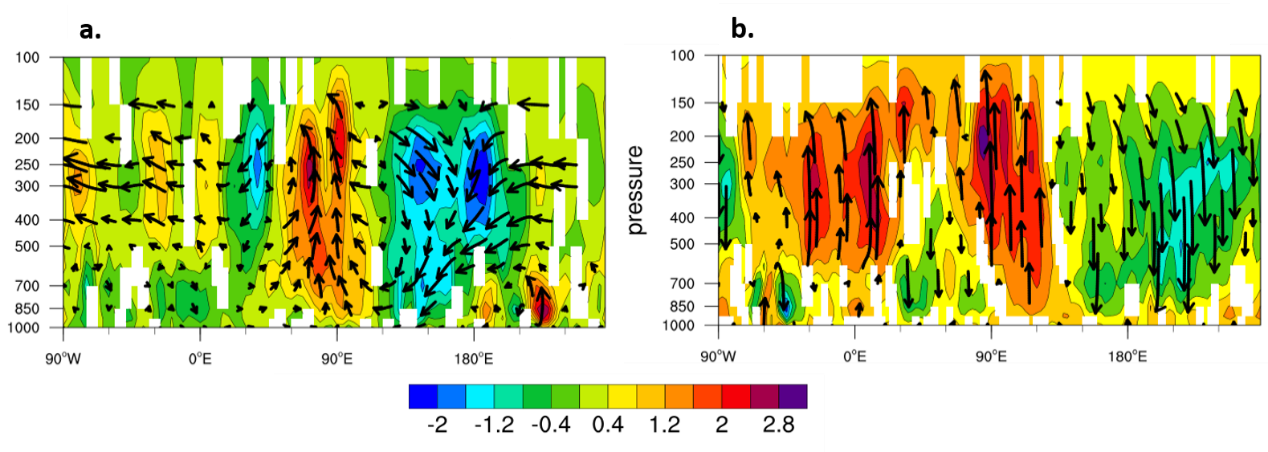


**Supplementary Figure 5 | Changes in atmospheric circulation from preindustrial (PI) simulation. a** Walker circulation changes (arrows) and troposphere vertical velocity anomalies (color shading) averaged between 5◦ S and 5◦ N over between PI_IO+1C and PI_IO+0C. The vertical velocity is magnified by a factor of 750 to make its scale comparable to that of zonal wind. **b** Same as (**a)** but between PI_NA+1C and PI_NA+0C.


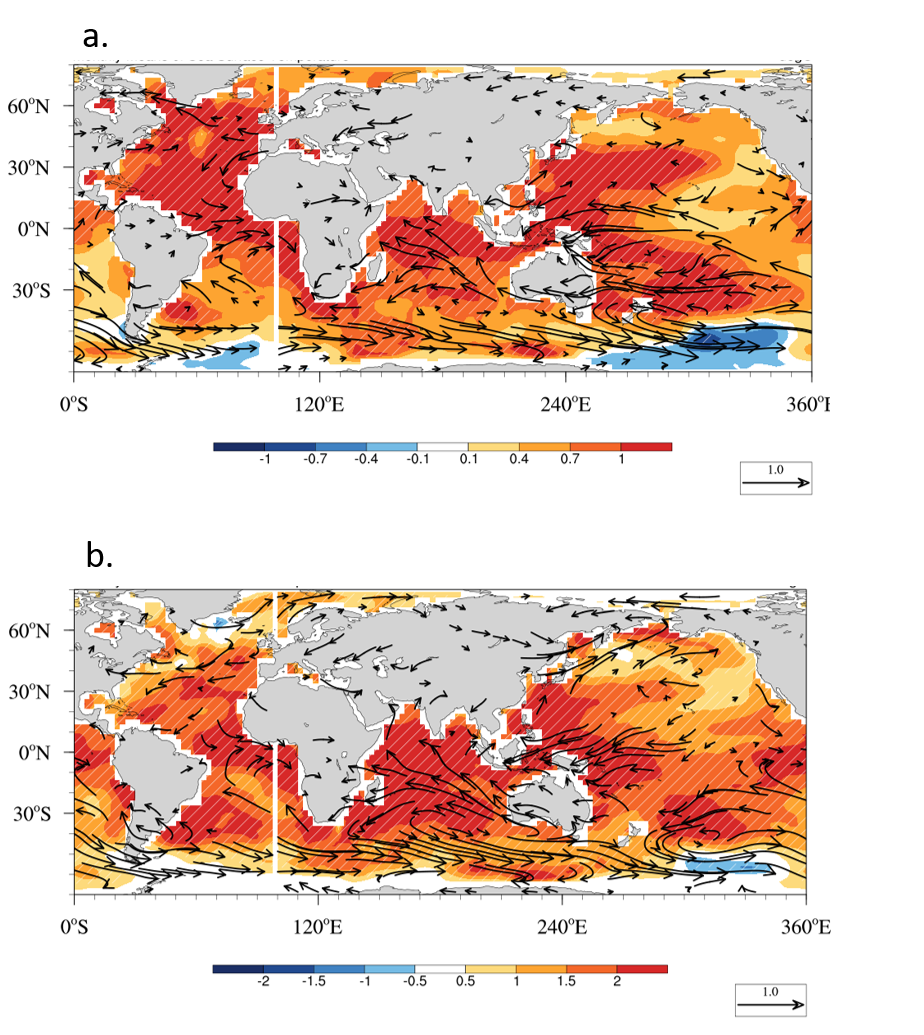


**Supplementary Figure 6 | Atmospheric response to North Atlantic Ocean (NA) and Indian Ocean (IO). a** SST (K) and surface wind anomalies (arrow, m/s) regressed onto the NA index from historical simulation using NESM3.0. The 11-year running average data were used for 1950–2020. The hatched area represents the regressed SSTA and is significant at the 95% confidence level. **b** Same as (**a)** but for the IO index.


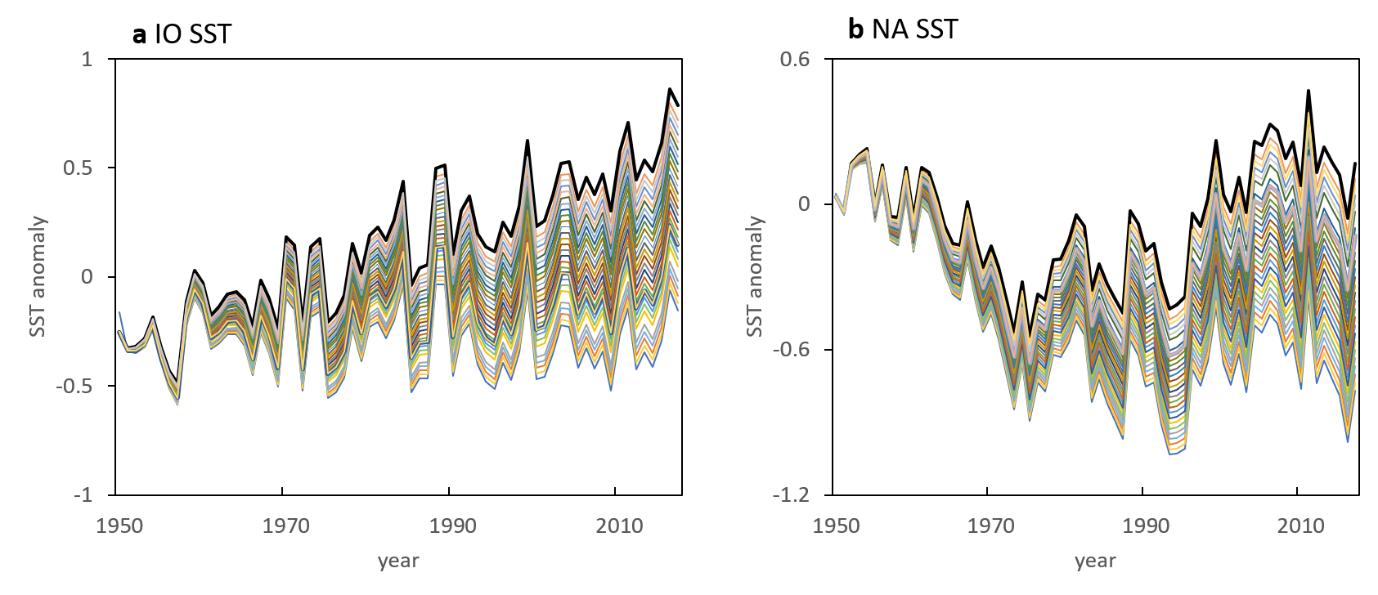


**Supplementary Figure 7 |** **Various SST forcings in historical simulations**. **a** Time series of annual mean observed IO SST. The black line shows observed SST anomalies and color lines represent IO SST with different trends. The trend of the black line is 0.0145C year-1 and the trends of each color line are monotonically reduced (interval is 0.05) and the trend of the bottom line is 0.01. **b** Same as (**a)** but for NA SST.


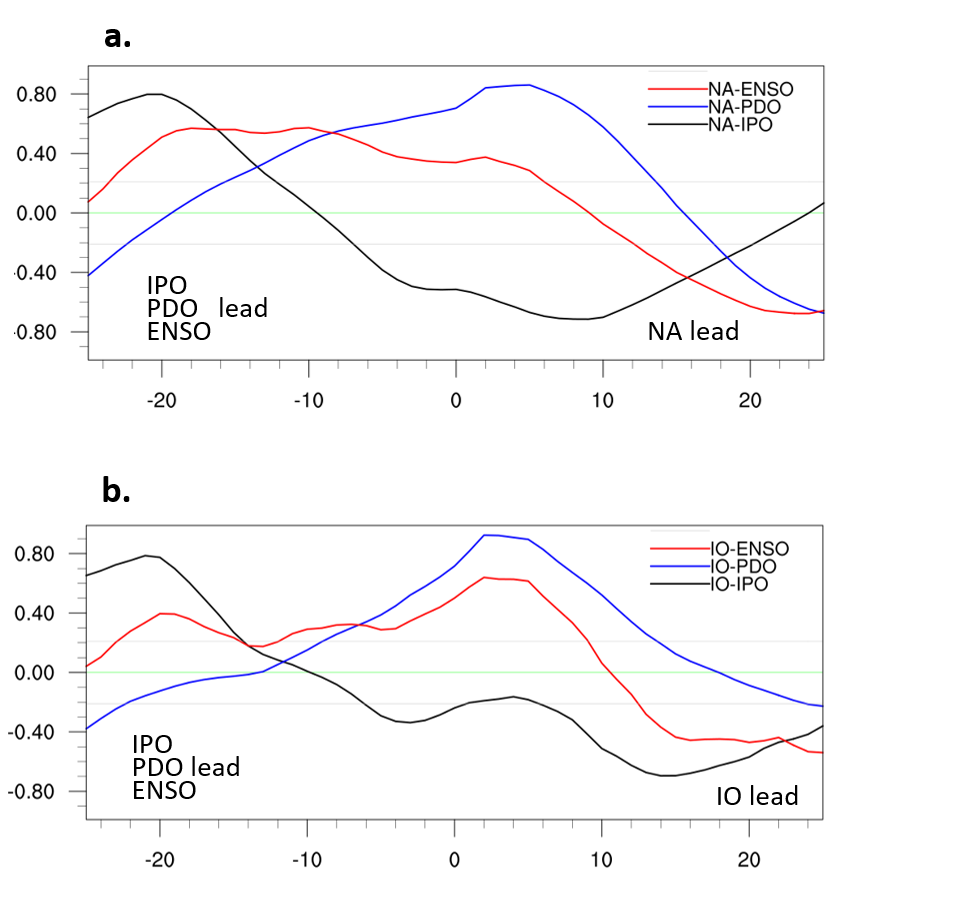


**Supplementary Figure 8 | Relationship between Indian Ocean (IO) and North Atlantic Ocean (NA) warming chain, and Pacific indices.** **a.** Observed lead-lag correlation coefficient between the NA and PDO, IPO, and decadal ENSO indices. The lag is positive (negative) when the NA leads (lags). PDO is defined as the leading principal component of North Pacific monthly sea surface temperature variability. The IPO is defined as the second EOF (after the global warming mode) of decadally low-pass filtered SST. The decadal ENSO is defined as the Nino3.4 index of decadally (11year) low-pass filtered SST. The grey lines represent a 95% significance level and the green line shows zero. **b** Same as **(a)** but for IO index**.** The 11-year running average data were used for 1950–2020 and long-term linear trends were removed. The last 5 years were excluded from the analysis and the long-term linear trends in the SST data were removed before regression.


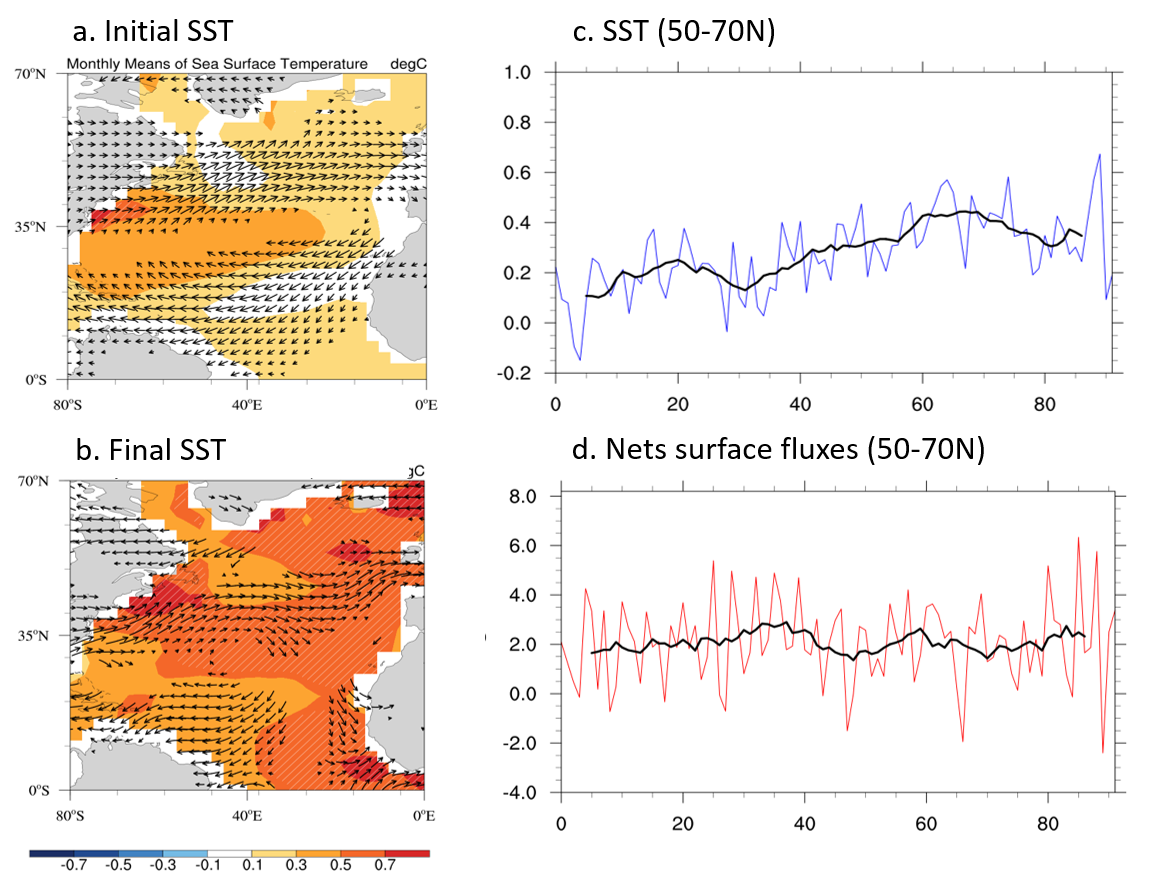


**Supplementary Figure 9 | North Atlantic response to Indian ocean warming**. **a** Horizontal patterns of SST anomalies between IO+0 and IO + 1C model experiments during the initial phase (0-30yrs). **b** Same as (**a**) but for the final phase (70-100yrs). **c** temporal evolution of SST anomalies averaged over 0°-80°W, 50°-70°N between PI_IO+0 and PI_IO + 1C model experiments. **d** Same as (**c**) but for net surface fluxes (W m^-2^). “Initial” refers to an average for Years 1–30, while “final” refers to Years 70–100. The blue (or red) line shows annual mean data and the black line represents an 11-year running mean of the red line (or blue line)


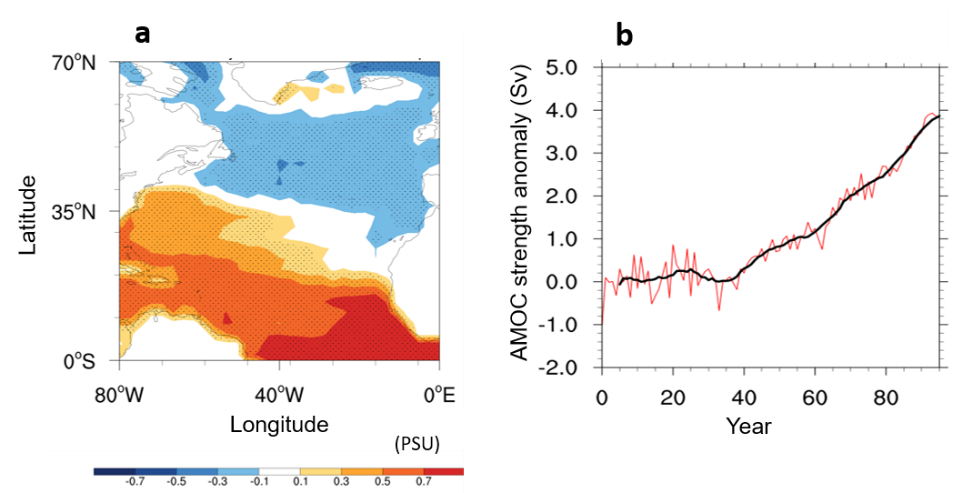


**Supplementary Figure 10 |** **Response of oceanic processes over North Atlantic Ocean (NA) region by Indian Ocean (IO) warming from pre-industrial simulations**. **a** Anomalies in Sea surface salinity (SSS) (PSU) from the fixed preindustrial (PI) simulation with observed IO SST anomalies. The anomalies are computed for the 100 years of the PI_IO+1C experiment with respect to the pre-industrial simulation (PI_IO+0, see Methods). The dotted area represents the regressed SSTA and is significant at the 95% confidence level. **b** Temporal evolution of anomalies in AMOC strength (Sv). 100-year simulated data are used for analysis. The AMOC strength is estimated as the maximum stream function within 500–5500 m, 30°‒70° N.
